# PGC-1α Inhibits Oleic Acid Induced Proliferation and Migration of Rat Vascular Smooth Muscle Cells

**DOI:** 10.1371/journal.pone.0001137

**Published:** 2007-11-07

**Authors:** Yan Zhang, Chang Liu, Lingyun Zhu, Xiaohong Jiang, Xi Chen, Xiaoqiang Qi, Xiangying Liang, Sonia Jin, Peixiang Zhang, Qingguo Li, Dongjin Wang, Xiaofeng Liu, Ke Zeng, Junfeng Zhang, Yang Xiang, Chen-Yu Zhang

**Affiliations:** 1 Jiangsu Diabetes Center, State Key Laboratory of Pharmaceutical Biotechnology, School of Life Sciences, Nanjing University, Nanjing, People's Republic of China; 2 Massachusetts Institute of Technology, Cambridge, Massachusetts, United States of America; 3 Department of Thoracic Surgery, The Affiliated Drum Tower Hospital of Nanjing University Medical School, Nanjing, People's Republic of China; Monash University, Australia

## Abstract

**Background:**

Oleic acid (OA) stimulates vascular smooth muscle cell (VSMC) proliferation and migration. The precise mechanism is still unclear. We sought to investigate the effects of peroxisome proliferator-activated receptor gamma (PPARγ) coactivator-1 alpha (PGC-1α) on OA-induced VSMC proliferation and migration.

**Principal Findings:**

Oleate and palmitate, the most abundant monounsaturated fatty acid and saturated fatty acid in plasma, respectively, differently affect the mRNA and protein levels of PGC-1α in VSMCs. OA treatment resulted in a reduction of PGC-1α expression, which may be responsible for the increase in VSMC proliferation and migration caused by this fatty acid. In fact, overexpression of PGC-1α prevented OA-induced VSMC proliferation and migration while suppression of PGC-1α by siRNA enhanced the effects of OA. In contrast, palmitic acid (PA) treatment led to opposite effects. This saturated fatty acid induced PGC-1α expression and prevented OA-induced VSMC proliferation and migration. Mechanistic study demonstrated that the effects of PGC-1α on VSMC proliferation and migration result from its capacity to prevent ERK phosphorylation.

**Conclusions:**

OA and PA regulate PGC-1α expression in VSMCs differentially. OA stimulates VSMC proliferation and migration via suppression of PGC-1α expression while PA reverses the effects of OA by inducing PGC-1α expression. Upregulation of PGC-1α in VSMCs provides a potential novel strategy in preventing atherosclerosis.

## Introduction

Pathogenic development of atherosclerosis involves a complex series of events [1], of which abnormal proliferation and migration of vascular smooth muscle cells (VSMCs) contribute significantly to the progression of atherosclerosis [2]. One independent risk factor for atherosclerosis is obesity [3], [4]. Obese hypertensive elevates plasma free fatty acids (FFAs) [5], particularly oleic acid (OA) [6], which promotes VSMCs from contractile to synthetic type, stimulates VSMC proliferation and migration to subendothelium, and contributes to the formation of organized atherosclerotic plaque [7]. OA, the most abundant unsaturated fatty acid in plasma [8], induces VSMC proliferation and migration by direct induction of extracellular signal-regulated kinase (ERK)-dependent mitogenic response [6], [9]–[11].

Recent studies demonstrated that thiazolidinediones (TZDs), the ligands for nuclear receptor peroxisome proliferator-activated receptor gamma (PPARγ), improved cardiovascular risk factors via exerting direct effects on vascular cells, for example, inhibition of VSMC proliferation and migration [12], [13]. It has been shown that TZDs inhibit key steps in the ERK/MAPK pathway, block the events that are critical for the re-entry of quiescent VSMCs into cell cycle, thus retard serum-induced growth of cultured arterial VSMCs and PDGF-BB-directed migration of VSMCs [13]–[15].

PPARγ coactivator-1 alpha (PGC-1α) is originally identified as a transcriptional coactivator of PPARγ [16]. Recent studies show that PGC-1α regulates the activity of several nuclear receptors and other transcriptional factors [17]. The basic biochemical function of PGC-1α is the regulation of mitochondrial biogenesis [18]. PGC-1α is widely expressed, including brown fat, skeletal muscle, liver, heart, kidney and brain [16], [19], [20]. PGC-1α plays important roles in many tissues, including the regulation of adaptive thermogenesis in brown fat and muscle [16], of muscle fiber-type switching in muscle [21], of gluconeogenesis in liver [22], and of insulin secretion in islets [23]. There are also large body of work to study regulation of PGC-1α expression in different tissues [24], [25]. It has been reported that elevated FFAs regulate expression of PGC-1α [26], [27], and certain FFAs affect PGC-1α expression differently [28]–[31]. Recently, Hondares *et al* have reported an autoregulatory loop between PGC-1α and PPARγ in adipocytes [32]. However, there is no report of regulation and function of PGC-1α in VSMCs.

In the present study, we examined the effects of FFAs (OA and/or palmitic acid PA) on PGC-1α expression in VSMCs. We also assessed the possible role of PGC-1α in OA-induced VSMC proliferation and migration. We found that OA and PA had an opposite role in regulating PGC-1α expression in rat VSMCs and PGC-1α inhibited OA-induced VSMC proliferation and migration via the inhibition of ERK phosphorylation.

## Results

### OA stimulated VSMC proliferation and migration which is associated with decreased PGC-1α expression

**Figure 1:**
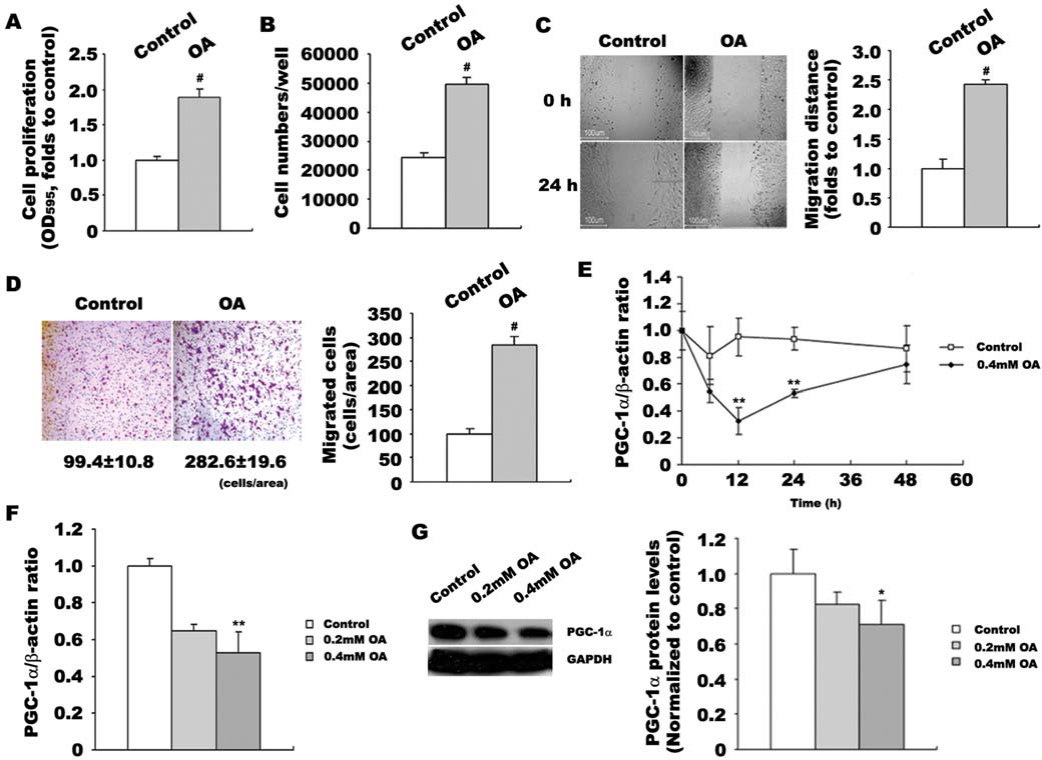
OA-stimulated VSMC proliferation and migration is associated with decreased expression of PGC-1α. VSMCs were incubated with 0.4 mmol/L OA for 24 h before analysis. VSMC proliferation were determined by MTT assay (A) and direct cell counting (B). VSMC migration was assessed by wound healing assay (C) and transwell analysis (D). The time course of 0.4 mmol/L OA effect on PGC-1α expression was linear over 48 h. PGC-1α expression was quantified by quantitative PCR (E). VSMCs were treated with various concentrations of OA for 24 h. PGC-1α expression was examined by quantitative PCR (E) and western blot (F). Data in VSMC proliferation and migration detections represent the means±SEM of 18 determinants from 3 independently prepared samples each with 6 measurements. Data in PGC-1α mRNA level detections are expressed as means±SEM of five different experiments normalized to β-actin levels. Data in PGC-1α protein level detections are expressed as means±SEM of four different experiments normalized to GAPDH levels. *P<0.05, **P<0.01, #P<0.001 vs. control.

OA was suggested to be the acting component of FFAs inducing VSMC proliferation and migration [6], [9]–[11]. In this study, we used 0.4 mmol/L OA to stimulate cultured rat VSMCs and detected changes of cell proliferation and migration. MTT assay showed that OA increased VSMC proliferation by 2-fold after 24 h stimulation (p<0.001 versus control, Fig. 1A). Direct cell counting confirmed that the increment in cell number (seeded at 1.5×10^5^ cells/well) was greater in OA treated VSMCs (4.97±0.23×10^4^ versus 2.45±0.16×10^4^ cells/well, p<0.001 versus control, Fig. 1B). Directional migration of VSMC monolayers after mechanical wounding was next performed, and the data presented in Fig. 1C demonstrated that OA increased VSMC migration by 2.5-fold (160.2±5.7 versus 63.8±3.5 µm, p<0.001 versus control). Transwell chamber assay also showed that OA-treated VSMCs migrated 2.8-fold faster than control cells (282.6±19.6 versus 99.4±10.8, p<0.001, Fig. 1D).

As shown in Fig. 1E, VSMCs were treated with 0.4 mmol/L OA, and then PGC-1α mRNA levels were detected at various time. The time course was linear over 48 h, representing initial decrease and increase thereafter, and PGC-1α mRNA level was significantly decreased at 12h and 24h after OA administration (p<0.01 versus control, Fig. 1E). VSMCs were also treated with various concentrations of OA for 24 h and PGC-1α expressions were examined by quantitative PCR and western blot. Results showed that PGC-1α mRNA and protein expressions were dose-dependently down-regulated by 0.2 mmol/L and 0.4 mmol/L OA, and decreased to the lower level (mRNA level:53%, p<0.01 versus control, Fig. 1F; protein level: 68%, p<0.05 versus control, Fig. 1G) at 0.4 mmol/L OA treatment.

### Increased PGC-1α expression inhibits OA-induced VSMC proliferation and migration

**Figure 2:**
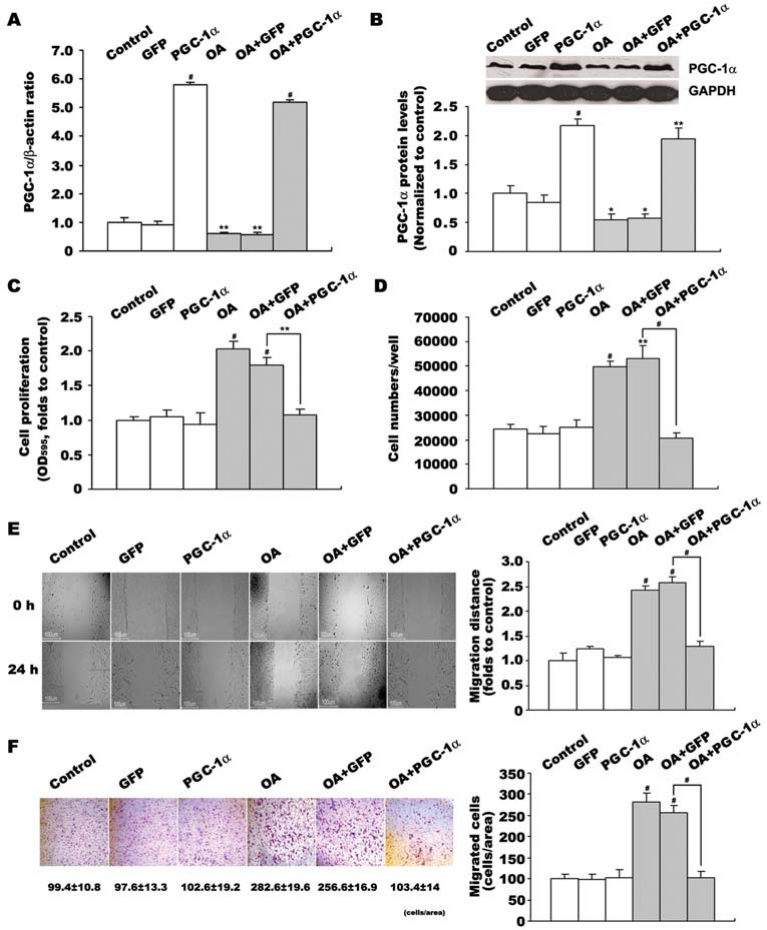
Effect of overexpressed PGC-1α on OA-induced VSMC proliferation and migration. VSMCs were treated with 48 h adenovirus infection, then incubated with 0.4 mmol/L OA for 24 h. PGC-1α expression was analysised by quantitative PCR (A) and western blot (B). Effects of PGC-1α overexpression on OA-induced VSMC proliferation were determined by MTT assay (C) and cell counting (D). Effects of PGC-1α overexpression on OA-induced VSMC migration were determined by wound healing (E) and transwell analysis (F). Data in VSMC proliferation and migration detections represent the means±SEM of 18 determinants from 3 independently prepared samples each with 6 measurements. Data in PGC-1α mRNA level detections are expressed as means±SEM of five different experiments normalized to β-actin levels. Data in PGC-1α protein level detections are expressed as means±SEM of four different experiments normalized to GAPDH levels. *P<0.05, **P<0.01, #P<0.001 vs. control or GFP group.

To investigate the role of PGC-1α in VSMC proliferation and migration, we stimulated PGC-1α expression by transient transfection with recombinant adenoviruses expressing PGC-1α/GFP protein. 48 h infection of Ad-PGC-1α markedly increased PGC-1α mRNA (over 5-fold, p<0.001 versus control, Fig. 2A) and protein (over 2-fold, p<0.01/0.001 versus control, Fig. 2B) levels in both quiescent and OA-stimulated VSMCs.

VSMC proliferation and migration were next analyzed by overexpressing PGC-1α. As shown in Fig. 2C–2F, OA still stimulated cell proliferation and migration in Ad-GFP infected VSMCs, suggesting that adenovirus infection with GFP alone had no effect on OA's stimulation. However, when cells were transfected with Ad-PGC-1α, which caused elevated PGC-1α expression, VSMC proliferation and migration were significantly decreased compared with control (Ad-GFP). MTT assay showed that VSMC proliferation decreased to 40% (p<0.01 versus Ad-GFP, Fig. 2C). Direct cell counting confirmed that the OA-induced increment in cell number was less in Ad-PGC-1α transfected VSMCs (2.06±0.24×10^4^ versus 5.32±0.29×10^4^ cells/well, P<0.001 versus Ad-GFP, Fig. 2D). At one time, OA-induced migration decreased to 31% (48.5±6.7 versus 155.7±4.7 µm, P<0.001 versus Ad-GFP, Fig. 2E). Transwell chamber assay also showed the number of migrated cells was decreased in Ad-PGC-1α transfected VSMCs (103.4±14.0 versus 256.6±16.9, P<0.001 versus Ad-GFP, Fig. 2F). PGC-1α almost inhibited OA-induced VSMC proliferation and migration to a quiescent level, but had no effect on quiescent VSMCs where OA was absent (Fig. 2).

### Suppression of PGC-1α by siRNA amplifies the OA-induced VSMC proliferation and migration

**Figure 3:**
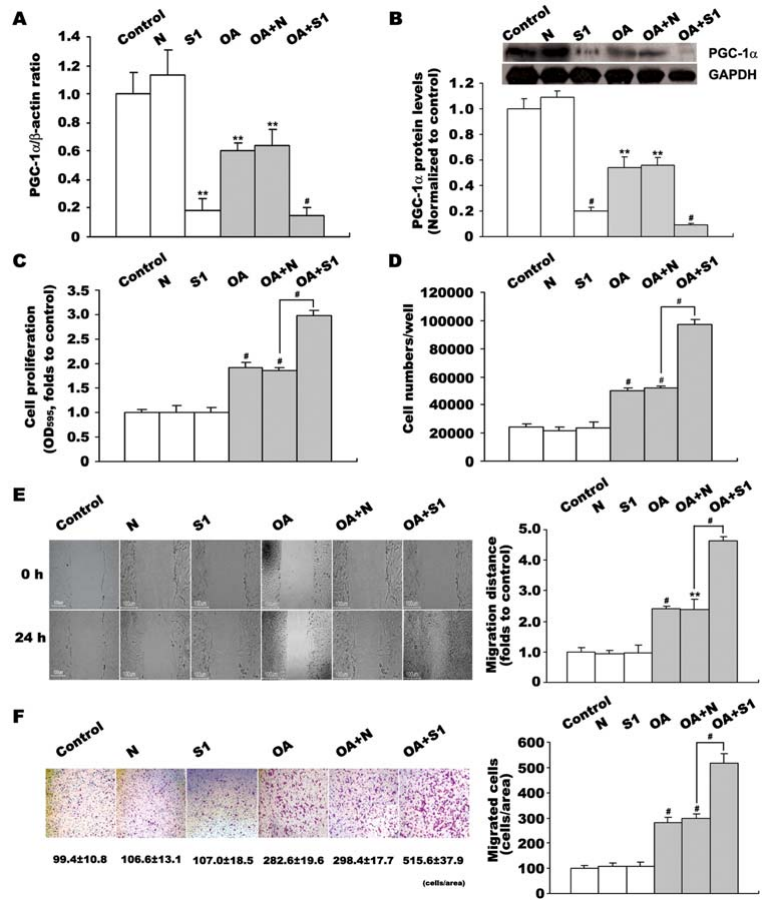
Effect of suppressed PGC-1α expression on OA-induced VSMC proliferation and migration. VSMCs were transfected with siRNA S1 or the negative control (siRNA N). The interference effect was assessed by quantitative PCR (A) and western blot (B). Effects of decreased PGC-1α on OA-induced VSMC proliferation were determined by MTT assay (C) and cell counting (D). Effects of decreased PGC-1α on OA-induced VSMC migration were determined by wound healing (E) and transwell analysis (F). Data in VSMC proliferation and migration detections represent the means±SEM of 18 determinants from 3 independently prepared samples each with 6 measurements. Data in PGC-1α mRNA level detections are expressed as means±SEM of five different experiments normalized to β-actin levels. Data in PGC-1α protein level detections are expressed as means±SEM of four different experiments normalized to GAPDH levels. **P<0.01, #P<0.001 vs. control or N group.

To further test our hypothesis that exogenous PGC-1α inhibits OA-induced VSMC proliferation and migration, we decreased PGC-1α expression by siRNA interference. As shown in Fig. 3A and 3B, siRNA S1 inhibited PGC-1α mRNA level by 80–88% (p<0.01/0.001 versus control) and protein level by 70%–80% (p<0.001 versus control) in both quiescent and OA-stimulated VSMCs. In contrast to the effects of elevated PGC-1α, the decrease of PGC-1α accelerated OA-induced VSMC proliferation and migration. OA-stimulated proliferation increased by 1.6-fold (p<0.001 versus N, Fig. 3C), and the cell number was greater in siRNA S1 transfected VSMCs (9.73±0.21×10^4^ versus 5.17±0.14×10^4^ cells/well, P<0.001 versus N, Fig. 3D). OA-induced directional migration increased by 1.8-fold (259.7±5.4 versus 143.6±5.7 µm, P<0.001 versus N, Fig. 3E), and the number of migrated cells was greater in siRNA S1 transfected VSMCs (515.6±37.9 versus 298.4±17.7, P<0.001 versus N, Fig. 3F). All these results demonstrated that suppression of PGC-1α amplified OA-induced VSMC proliferation and migration, but also had no effect on quiescent VSMCs (Fig. 3).

### PA induces endogenous PGC-1α expression and inhibits OA-induced VSMC proliferation and migration

**Figure 4:**
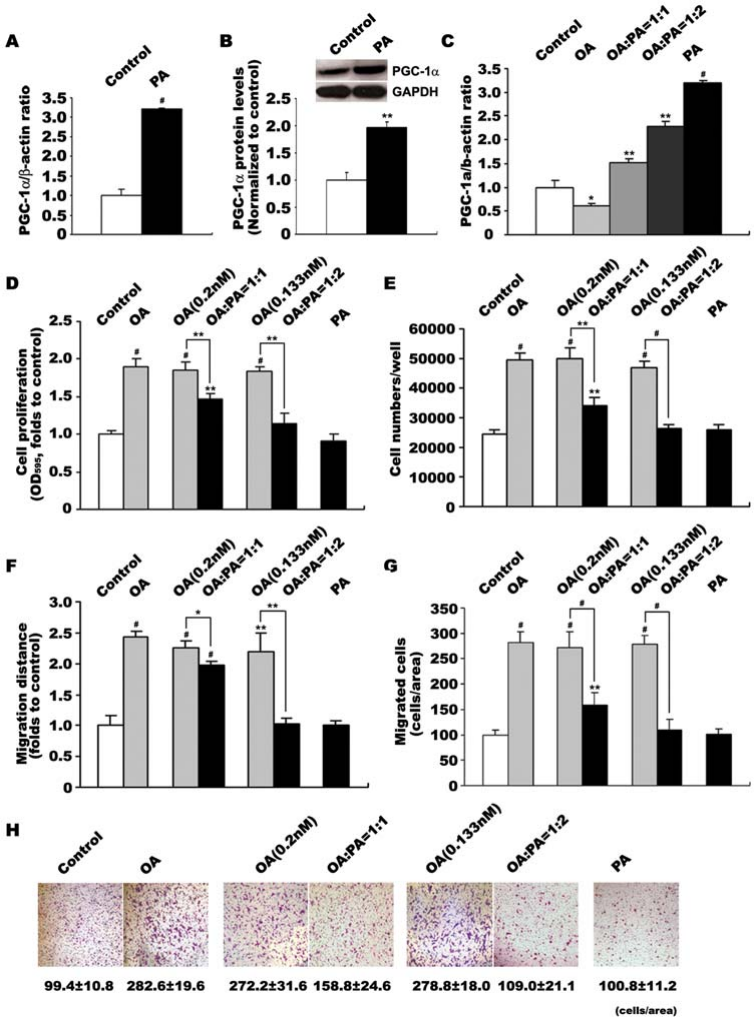
Changes of PGC-1α expression and VSMC proliferation/migration in response to increased proportion of PA in fatty acid mixtures. VSMCs were incubated with various FFAs for 24 h before analysis. Effects of PA on PGC-1α expression was determined by quantitative PCR (A) and western blot analysis (B). Effects of increased PA on PGC-1α expression were determined by quantitative PCR (C). Effects of increased PA on OA-induced VSMC proliferation were determined by MTT assay (D) and cell counting (E). Effects of increased PA on OA-induced VSMC migration were determined by wound healing (F) and transwell analysis (G,H). Data in VSMC proliferation and migration detections represent the means±SEM of 18 determinants from 3 independently prepared samples each with 6 measurements. Data in PGC-1α mRNA level detections are expressed as means±SEM of five different experiments normalized to β-actin levels. Data in PGC-1α protein level detections are expressed as means±SEM of four different experiments normalized to GAPDH levels. *P<0.05, **P<0.01, #P<0.001 vs. control

Palmitic acid (PA) is the most prevalent saturated FFA in circulation [33]. In this study, we also examined the effects of PA on VSMC growth and PGC-1α expression. In contrast to OA, addition of 0.4 mmol/L PA to VSMCs for 24 h markedly increased PGC-1α mRNA level by 3.2-fold (p<0.001 versus control, Fig. 4A) and protein level by 2-fold (p<0.01 versus control, Fig. 4B). So we used PA as an inducer of endogenous PGC-1α and further examined PGC-1α's functions in VSMCs.

As shown in Fig. 4C–4H, when OA and PA were mixed to a final concentration of 0.4 mmol/L, PGC-1α expression was upregulated in a dose-dependent manner with increasing PA fraction, and consequently led to the inhibition of OA-induced VSMC proliferation and migration. 1∶1 molar ratio of OA:PA moderately increased PGC-1α expression (Fig. 4C), but still stimulated VSMC proliferation and migration, although the stimulation was depressed compared with the effects of OA alone (Fig. 4D–4H); Increasing PA fraction in OA/PA mixture with ratio 1∶2 (0.133 mmol/L OA to 0.267 mmol/L PA) stimulated higher endogenous PGC-1α expression (Fig. 4C), and suppressed VSMC proliferation and migration to a quiescent level (Fig. 4D–4H). Please note that this quiescence was due to the increased amount of PA, but not due to the decreased amount of OA, because 0.133 mmol/L and 0.2 mmol/L OA still increased VSMC proliferation and migration as well as 0.4 mmol/L OA (Fig. 4D–4H).

### Effect of PGC-1α inhibition on p-ERK activity in response to OA

**Figure 5:**
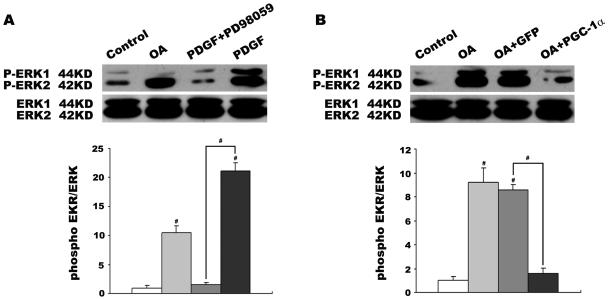
Effects of PGC-1α on OA-induced p-ERK activity in VSMCs. PDGF-BB and pharmacological ERK-MAPK inhibitor PD98059 were chosen as positive and negative controls of ERK phosphorylation, respectively. VSMCs were made quiescent by serum-starvation for 24 h and then stimulated with PDGF-BB (100 ng/ml) for 30 min, PD98059 (50 uM) for 1 h before stimulation with PDGF-BB (100 ng/mL), or 0.4 mmol/L OA for 2 h. Proteins extracted following these treatments and phosphorylation of ERK was determined by western blot (A). VSMCs were also treated with 48 h adenovirus infection and then incubated with 0.4 mmol/L OA for 24 h. Phosphorylation of ERK was also analyzed with elevated PGC-1α level by western blot (B). Data were shown as the ratio of p-ERK/total ERK. The ratios of control were designated as 1.0. Individual data in this chart represent the mean±SEM of 4 determinants. #P<0.001 vs. control or positive/negative group.

OA stimulated VSMC proliferation and migration through the activation of MAPK ERK signaling [6], [9]–[11]. To investigate whether ERK is activated by OA in VSMCs, we performed western blot analysis with a mouse monoclonal antibody that recognized the phosphorylated ERK (p-ERK). PDGF-BB (Sigma) and pharmacological ERK-MAPK inhibitor PD98059 (Cell signal) were chosen as positive and negative controls of ERK phosphorylation, respectively. VSMCs were made quiescent by serum-starvation for 24 h and then stimulated with PDGF-BB (100ng/ml) for 30 min, PD98059 (50uM) for 1 h before stimulation with PDGF-BB, or 0.4 mmol/L OA for 24 h. Western blot revealed the presence of p-ERK in VSMCs (Fig. 5). As shown in Fig. 5A, low amounts of p-ERK were found in 24 h serum-starved VSMCs. The level of p-ERK was significantly increased by PDGF-BB (over 20-fold, p<0.001 versus control), and this increase was inhibited 90% by PD98059 (p<0.001 versus PDGF alone). 0.4 mmol/L OA also increased the phosphorylation of ERK (over 10-fold, P<0.001 versus control) as well as PDGF-BB.

To study whether inhibitory effects of PGC-1α on VSMC proliferation and migration were related to MAPK ERK pathway, phosphorylation of ERK was also analyzed in VSMCs with elevated PGC-1α. VSMCs treated with 48 h adenovirus infection were incubated with 0.4 mmol/L OA for 24 h, and then p-ERK was detected by western blot. Results showed that OA still stimulated phosphorylation of ERK in Ad-GFP transfected VSMCs, however, when cells were transfected with Ad-PGC-1α, the phosphorylation of ERK decreased approximately 80% (p<0.001 versus Ad-GFP, Fig. 5B), which almost reverted the OA-induced phosphorylation of ERK, and accordingly inhibited OA-induced VSMC proliferation and migration.

## Discussion

PGC-1α, an important transcriptional coactivator, plays a key role in energy metabolism [16], [17]. PGC-1α is closely related to FFA metabolism, and its expression level changes in various tissues in rodent models of metabolic disorders [17], [25]. It has also reported that various saturated, unsaturated, and trans fatty acids, including monounsaturated (oleic acid, C_18∶1_) and polyunsaturated (linoleic acid, C_18∶2_; EPA, C_20∶5_; DHA, C_22∶6_; and arachidonic acid, C_20∶4_), saturated fatty acids of varying chain length (C_10∶0_ to C_18∶0_), and trans fatty acids such as elaidic acid (*trans*-C_18∶1_) and *trans-*vaccenic acid (*trans*-C_18∶1_) which are abundantly present in hydrogenated vegetable oil and dairy products, had no effect on the expression of PGC-1α, except stearic acid (C_18∶0_), induced PGC-1α mRNA 2.2-fold in primary hepatocytes [28]. Staiger H *et al* reported that PGC-1α expression was increased two- to three-fold by all unsaturated fatty acids they tested, while, saturated fatty acids did not modulate PGC-1α expression in human myotubes [29]. Coll T *et al* and Crunkhorn S *et al* have found that PA downregulated PGC-1α in myotubes [30], [31]. In the present study, we have found that OA inhibited, while PA stimulated PGC-1α expression in VSMCs (Fig. 1 and Fig. 4). Taking together, these results indicate that certain fatty acids regulate PGC-1α expression in a tissue-autonomous manner, and it may be related to the different physiological role of PGC-1α played in tissues.

Given the facts that PGC-1α played a key role in energy metabolism and that elevated FFAs level is one of major risk factors in many metabolic disorders, it is important to understand the mechanism of FFA regulating PGC-1α expression. Coll T *et al* described the MEK1/2 inhibitors PD98059 and U0126 prevented PA-induced downregulation of PGC-1α in C2C12 myotubes [30]. Furthermore, nuclear factor- kappaB (NF-κB) activation was also involved in palmitate-mediated PGC-1α downregulation [30]. Interestingly, Crunkhorn S *et al* have found that PA inhibiting PGC-1α expression required p38 mitogen-activated protein kinase activation in the same C2C12 myotubes [31]. In adipocytes, Hondares E *et al* demonstrated that there is an autoregulatory loop controls PGC-1α expression via PPARγ coactivation [32]. We have found that FFA (0.4 mmol/L, equimolar mixtures of OA and PA) induced PPARγ mRNA level in VSMCs, and downregulation of PGC-1α retarded the FFA's induction (Data not shown). Thus, it is interesting to further study whether the autoregulatory loop of PGC-1α-PPARγ is also existed in VSMCs.

It is well established that OA induces VSMC proliferation [9] and migration [11], plays a central role in obesity and high fatty acids induced atherosclerosis [7]. In this study, we have found that overexpression of PGC-1α by adenovirus infection blocked OA-induced VSMC proliferation and migration (Fig. 2). In contrast, suppression of PGC-1α expression by siRNA amplified the OA's effects on VSMCs (Fig. 3). Interestingly, PGC-1α had no effect on quiescent VSMCs where OA was absent (Fig. 2 and Fig. 3). Combined together, the results revealed that PGC-1α is an important negative regulator for VSMC proliferation and migration under pathophysiological conditions, such as OA stimultion. PA is the most prevalent saturated FFA in circulation [33]. In contrast to OA, PA markedly induced PGC-1α expression in VSMCs (Fig. 4B). Decrease of OA/PA ratios caused dose-dependent stimulation of PGC-1α expression and gradually reversed the OA's effects on VSMC proliferation and migration (Fig. 4C–4H). Furthermore, downregulation of PGC-1α by RNAi blocked the effects of PA (Data not shown). These results demonstrated the induction of endogenous PGC-1α by PA also inhibited OA-induced VSMC proliferation and migration, which further confirmed the negative role of PGC-1α in VSMC growth. Of note, 1:1 molar ratio of OA/PA with a final concentration of 0.4 mmol/L mimics the high fatty acid pathophysiological state [34], [35]. It moderately increased PGC-1α expression but still stimulated VSMC proliferation and migration, suggesting that increase of PGC-1α by pathophysiological fatty acid maybe not sufficient to retard OA-induced VSMC proliferation and migration.

The mechanism of OA-induced VSMC proliferation and migration has been proposed based on several facts: 1, OA directly induced mitogenesis in cultured VSMCs through increasing ERK phosphorylation [6], [9]–[11], an active form of ERK (p-ERK), which in turn, promoted VSMC proliferation and migration; 2, ERK1/2 was an upstream regulator in mitogenic response and was activated by agonists for tyrosine kinase encoded receptors and G protein-coupled receptors that initiated mitogenesis or cellular differentiation [36]; 3, ERKs mediated the effects of these agonists by phosphorylating and regulating the activity of a number of proteins and transcription factors, such as Elk-1 and Ets-1 [36], [37], which evoked c-Fos and MMP-9 [37], [38], and contributed to VSMC proliferation and migration, respectively. In the present study, we demonstrated that OA markedly increased phosphorylated ERK1/2 protein levels in VSMCs, whereas overexpression of PGC-1α almost abolished this stimulation of p-ERK (Fig. 5). These data indicate that PGC-1α inhibits OA-induced p-ERK activity, consequently, inhibits VSMC proliferation and migration.

The mechanism by which fatty acids (OA and PA) affect PGC-1α mRNA and protein levels, however, remains unknown. Previous studies show that the PPAR family of proteins may be involved in the process of development of atherosclerosis [39], [40]. TZD, a PPARγ ligand, inhibits VSMC proliferation and migration through the activation of PPARγ, which tends to inhibit the expression of several genes involved in extracellular signal-regulated kinase (ERK)-dependent mitogenic response, leading to the inhibition of cell growth and, finally, cell migration [12]–[15]. Furthermore, Hondares E *et al* demonstrated that there is an autoregulatory loop controls PGC-1α expression via PPARγ coactivation in adipocytes [32]. Given that PGC-1α acts as a transcriptional coactivator for PPARγ, it is very possible that the inhibition of PGC-1α in OA-induced VSMC proliferation and migration is PPARγ dependent. In fact, we have found that overexpression of PGC-1α induced PPARγ mRNA expression in quiescent VSMCs. Downregulation of PGC-1α retarded FFA (0.4 mmol/L, equimolar mixtures of OA and PA) induced PPARγ mRNA level (Data not shown). It has been described that TZD actives PPARγ, arrests MAPK ERK pathway [39], [40], [12]–[15], thus, prevents c-fos production which is required for S-phase entry induced in VSMCs [41], and inhibit MMP-9 production which is required in VSMC migration [42]. We have also found that PGC-1α negatively regulate not only p-ERK but also c-fos/MMP-9 expressions in OA-stimulated VSMCs (Data not shown). Taking together, PGC-1α increases PPARγ expression, and negatively regulates ERK MAPK pathway, consequently inhibits OA-induced VSMC proliferation and migration. Moreover, PPARγ RNAi or antagonist should be used for assessing the precise mechanism of PGC-1α affecting OA-induced VSMC proliferation and migration in the PPARγ-dependent manner in further study.

In summary, we demonstrated that OA and PA regulate PGC-1α expression differently in VSMCs. OA stimulates VSMC proliferation and migration via suppression of PGC-1α expression. Upregulation of PGC-1α in VSMC could be a novel strategy of treatment of atherosclerosis.

## Materials and Methods

### Cell culture

Rat aortic VSMCs were isolated from the thoracic aortas of 3- to 4-week-old male Sprague-Dawley rats as described previously [43]. Isolated VSMCs were cultured in DMEM (Gibco-Invitrogen, Carlsbad, USA) supplemented with 10% FCS (GBICO BRL, Rockville, MD), 25 mmol/L HEPES (pH 7.4), penicillin (100 U/mL), and streptomycin (100 µg/mL) at 37°C in a humidified atmosphere of 95% air and 5% CO_2_. VSMCs were identified by the characteristic “hill and valley” growth pattern and positive immunocytochemical staining with a monoclonal antibody against smooth muscle α-actin (Progen). Cells after the 4th to 8th passages were used in experiments.

### Adenovirus infection

Recombinant adenoviruses expressing PGC-1α-GFP (Green Fluorescence Protein) protein and GFP alone were provided by Dr. Dan Kelly (Washington University, Saint Louis, MO, USA). Purified virus stocks were prepared through CsCl step gradient centrifugation as described previously [44]. Cells were grown to confluence, induced to differentiate for 48 h, followed by infection with adenovirus at multiplicities of infection (moi) of 50. The infection efficiency was determined by fluorescence intensity of GFP. Cells were harvested for total RNA extraction after 48 h infection.

### siRNA interference

To knock down PGC-1α expression in VSMCs, three siRNA sequences targeting different sites of rat PGC-1α cDNA were designed and synthesized by Genesil (Wuhan, P.R. China), including a control sequence which could not target PGC-1α cDNA. siRNA was transfected into VSMCs using LipofectAmine reagent (Gibco-Invitrogen) according to the manufacture's manual. 48 h after medium replacement, VSMCs were harvested for total RNA isolation, and PGC-1α expression level was analyzed by quantitative PCR. The sequence with the best interfering effect (named S1) was selected. siRNA sequences were as follows: S1, AAGACGGATTGCCCTCATTTG; S2, AAGAGCCGTCTCTACTTAAGA; S3, AAGCTTGCGCAGGTAACATGT; N (the negative control sequence), AAGCTTCATAAGGCGCATAGC.

### Fatty acid treatments on VSMCs

OA and PA (Sigma) were dissolved in ethanol as 50 mmol/L stock solutions respectively, and were further diluted in DMEM medium supplemented with 2% fatty acid free BSA (Sigma) to a final concentration of 0.4 mmol/L. The 0.4 mmol/L OA/PA mixtures with molar ratios of 1∶1 and 1∶2 were also prepared. FFAs were applied to VSMCs for 24 h before analysis.

### VSMC Proliferation Assays

The microculture tetrazolium (MTT) assay was used to detect VSMC proliferation [45]. Cells seeded in 96-well plates (1×10^4^ cells/well) were cultured up to confluence, and the medium was replaced by fresh serum-free medium. The cells were then treated with adenovirus infection or siRNA interference for 48 h, followed by 24 h fatty acids stimulation. At the end of this period, MTT (0.2 mg/mL) was added to each well, and incubation proceeded for 4 h at 37°C. Thereafter, the culture medium was removed and the cells were solubilized in 100 mL DMSO. The extent of reduction of MTT to formazan within cells was quantified spectrophotometrically at 595 nm and taken as an indicator of cell viability [45]. Cell counting analysis was used to confirm the MTT analysis. VSMCs were seeded in 6-well plates (1.5×10^5^ cells/well) and treated in the same way. Cells were then resuspended with 0.05% trypsin and 0.02% EDTA, and cell numbers were determined by a hemocytometer.

### VSMC migration assays

Migration assays were performed by 2 approaches: scratch wound motility assay [46], [47] and the modified Boyden chamber [48], [49]. For the scratch wound motility assay, VSMCs were seeded in 6-well plates (1.5×10^5^ cells/well) and grew to confluence. 24 h after serum deprivation, VSMCs were treated with adenovirus infection or siRNA interference for 48 h. Treated cells were then mounted to a reusable template to create a standard wound (≈3 mm), followed by 24 h fatty acids treatment. Wound closure rates were followed with a reference point in the field of the wound at the bottom of the plate by direct microscopic visualization. The procedure permitted photographing the identical spot each time. The remaining cell-free area was determined via microphotography and performed immediately after 24 h injury. Differences were analyzed using NIH Image 1.6 program (Wayne Rasband). Boyden chamber cell migration assay was performed using transwell chambers with fibronectin (Sigma)-coated 8-μm-pore-size polycarbonate membranes (BD Biosciences). Preconfluent VSMCs treated in the same way were then suspended in DMEM-0.5% FBS to a concentration of 4×10^5^ cells/mL. Different fatty acids or serum free DMEM (0.6 mL) were added to the lower compartment. A 0.1-mL cell suspension (final concentration, 4×10^4^ cells/well; diameter, 6.5 µm) was added to the upper compartment, and cells were then incubated at 37°C (95% air-5% CO_2_). 6 h later, nonmigrated cells were removed with a cotton swab, and the migrated cells were fixed with paraformal-dehyde for 30 min and stained with crystal violet. Cell migration was quantified by blind counting of the migrated cells on the lower surface of the membrane of 5 fields per chamber under microscope.

### RNA analysis

To examine the expression level of PGC-1α in rat VSMCs, total RNA was isolated using RNeasy kit (Qiagen, Hilden, Germany). One microgram of RNA was reverse-transcribed into cDNA with oligo (dT)_18_ primers and AMV reverse transcriptase (Gibco invitrogen, Carlsbad, CA, USA) at 42°C for 1 h. Quantitative PCR was performed with the ABI Prism 7000 sequence detection system (Applied Biosystem, Foster City, CA) with Tagman probes (Shanghai Shinegene Molecular Biotechnology Co., LTD.), and threshold cycle numbers were obtained using ABI Prism 7000 SDS software version 1.0. Amplification conditions were: one cycle of 95°C for 5 min followed by 40 cycles of 95°C for 30 s, 60°C for 1 min, and final one cycle of 72°C for 2 min. Sequences of quantitative PCR primers and probes were as follows: rat PGC-1α, 5′-AGGTCCCCAGGCAGTAGAT-3′ (sense), 5′-CGTGCTCATTGGCTTCATA-3′ (antisense), 5′FAM+ATGAATCAAGCCACTACAGACACC+TAMRA3′ (probe); rat β-actin, 5′-AGGGAAATCGTGCGTGAC-3′ (sense), 5′-CGCTCATTGCCGATAGTG-3′ (antisense); 5′ FAM+CTGTGCTATGTTGCCCTAGACTTC+TAMRA3′ (probe).

### Western blot analysis

To obtain total proteins, VSMCs were lysesed in a buffer containing 10 mmol/L HEPES (pH 7.9), 1.5 mmol/L MgCl_2_, 10 mmol/L KCl, and 0.5% NP-40, and cell lysates were centrifuged at 13000 g for 5 min. Supernatants were collected as cytosolic extracts for western blot. Nuclear extracts were isolated according to Andrews *et al* [50]. Protein concentration was determined colorimetrically with BCA method (Pierce, Rockford, USA). Nuclear extracts containing 200 µg nuclear proteins were applied to 10% SDS-PAGE gel electrophoresis for PGC-1α detection, and rabbit polyclonal PGC-1α antibody (Santa Cruz Biotechnology, Santa Cruz, USA) was used as primary antibody. Samples containing 30 µg total proteins were applied to 15% SDS-PAGE gel electrophoresis for phosphorylated ERK1/2 detection. Phospho-specific mouse anti-human ERK1/2 antibody (Santa Cruz Biotechnology, Santa Cruz, USA), which detected only phosphorylated activated forms, and mouse anti-human ERK antibody (Santa Cruz) were used as primary antibody. After incubation with primary antibodies for 2 h and washing, membranes were incubated with corresponding horseradish peroxidase (HRP) conjugated secondary antibody, and detected with a chemiluminescent detection system (NEN, Boston, USA).

### Statistical analysis

All results are expressed as means±SEM. Data were analyzed using a one-way ANOVA followed by Fisher's LSD post-hoc test. Calculations were performed using SPSS/Windows version 12.5S statistical package (SPSS, Chicago, IL, USA). In all cases P≤0.05 was taken as statistically significant.
